# The Impact of Ignoring a Crossed Factor in Cross-Classified Multilevel Modeling

**DOI:** 10.3389/fpsyg.2021.637645

**Published:** 2021-03-03

**Authors:** Soyoung Kim, Yoonhwa Jeong, Sehee Hong

**Affiliations:** ^1^Institute of Educational Research, Korea University, Seoul, South Korea; ^2^Talent Development Group, Samsung Electronics Leadership Center, Yong-in, South Korea; ^3^Department of Education, Korea University, Seoul, South Korea

**Keywords:** cross-classified random effect modeling, multilevel data, feeder, magnitude of coefficients, crossed factor, Monte-Carlo simulation study

## Abstract

The present study investigated estimate biases in cross-classified random effect modeling (CCREM) and hierarchical linear modeling (HLM) when ignoring a crossed factor in CCREM considering the impact of the feeder and the magnitude of coefficients. There were six simulation factors: the magnitude of coefficient, the correlation between the level 2 residuals, the number of groups, the average number of individuals sampled from each group, the intra-unit correlation coefficient, and the number of feeders. The targeted interests of the coefficients were four fixed effects and two random effects. The results showed that ignoring a crossed factor in cross-classified data causes a parameter bias for the random effects of level 2 predictors and a standard error bias for the fixed effects of intercepts, level 1 predictors, and level 2 predictors. Bayesian information criteria generally outperformed Akaike information criteria in detecting the correct model.

## Introduction

Hierarchical linear modeling (HLM) can be used when the levels in a multilevel data structure are strictly nested. The HLM technique is a fairly common analysis method in educational settings (e.g., students are nested within schools). In the case of purely hierarchical data structures, lower-level entities are nested into only one higher-level entity (Raudenbush and Bryk, 2002). However, behavioral scientists frequently encounter cross-classified data structures, i.e., there are multiple sources of membership for lower-level entities (Meyers and Beretvas, 2006). For example, in the field of clinical and medical treatment, patients could have multiple sources of membership, such as doctors or nurses, while in the field of education, students could have multiple sources of membership, such as high schools and hometowns. HLM, which is purely a nested multilevel model, requires extending to reflect cross-classified multilevel data structures.

Cross-classified random effect modeling (CCREM) includes two or more multilevel data structures due to lower-level entities' multiple sources of memberships and has arisen from the prevalence of cross-classified datasets (Hox, 1998; Ecob et al., 2004; Hough, 2006; Ye and Daniel, 2017). In large-scale panel surveys, cross-classifications are common (Chung et al., 2018, Luo and Kwok, 2009). For example, in longitudinal surveys for children and adolescents within educational research, students' affiliations shift upon their entrance to middle or high school. When students' achievement is affected by both their middle school and high school variables, there are two crossed factors in the upper-level structure. If this data structure is not reflected in the analysis, the results could be misleading.

Researchers have pointed out the problem of the misspecification of a crossed factor in cross-classified data (Meyers and Beretvas, 2006; Luo and Kwok, 2009; Ye and Daniel, 2017). Meyers and Beretvas (2006) compared HLM and CCREM and found that the standard error (SE) is biased in HLM when the parameters are generated under CCREM. The estimate of the random component of the remaining factor was overestimated when a crossed factor was ignored. They also found that Bayesian information criteria (BIC) identified CCREM as the correct model under all conditions. Ye and Daniel (2017) added a random slope to Meyers and Beretvas' (2006) study and found that the SE of the misspecified level 1 predictor's regression weight was underestimated in HLM when the predictor originated from the level 2 variable. Luo and Kwok (2009) examined a three-level CCREM with crossed factors at the top and intermediate levels and revealed that the SE of fixed effects was underestimated at the upper level and overestimated at the lower level when a crossed factor was ignored at the upper level. For predictors that are not related to the omitted crossed factor, the regression weight under HLM did not show significant biases.

The simulation studies mentioned above have two limitations for realistic application. First, the effect sizes of the coefficients were set to .50 in the previous studies. Effect size, here, denotes the magnitude of the coefficient, and fixing a coefficient at .50 means the coefficient has a moderate effect size. However, small effect sizes commonly occur in the field of education (Spybrook, 2008). As effect size affects statistical power along with sample size (Meuleman and Billiet, 2009), effect size and sample size should be considered together. Thus, effect size, which is the magnitude of a coefficient, needs to be considered as a condition to make research more useful in the real world.

Second, researchers have investigated the influence of the number of feeders on bias, but the results have been controversial. The feeder is information about the data structure of multiple memberships in CCREM. The more feeders, the more membership affiliations overlap with each other, making the data structure more cross-classified. Meyers and Beretvas (2006) manipulated the number of feeders from two to three and concluded that the number of feeders did not affect the estimate bias. However, other studies have produced contradictory results. Luo and Kwok (2009) manipulated the number of feeders and noted that cross-classified data structures influenced bias. Lee and Hong (2019) also manipulated the number of feeders from two to six and concluded that the coefficient of the CCREM interaction term is affected by the number of feeder conditions.

Several previous studies have examined the effects of the simulation factors we considered in this study. For example, Lai (2019) considered the number of clusters at each cross-classified factor, the degrees of imbalance, and cell sizes, and Ye and Daniel (2017) examined the slope of level 1 predictors, the relationships between level-2 residuals, the sample sizes of each level, and the magnitudes of intra-class correlation. However, given that all the relevant simulation conditions were not evaluated simultaneously in an integrated manner, it was difficult to understand the interaction effects among the simulation factors. It could be possible that there were significant main effects of each simulation condition, but the significance of effect could be changed when conditions are joined at the same time due to the interaction. We argue that our study makes unique contributions at this point by simultaneously considering the main and interaction effects of all the relevant simulation conditions.

The purpose of the current study is to compare the statistical performance of CCREM and HLM when the correct model is generated by CCREM considering the magnitude of the coefficients and the number of feeders. Estimate bias and SE bias are investigated to ascertain the impact of ignoring a crossed factor in a cross-classified multilevel analysis, and the accuracy of information criteria indices is also examined. As there may be real-world situations where it is better to use HLM instead of CCREM due to survey design or data collection status (e.g., neighborhood ID is missing after collecting data), the degree of estimate bias requires investigation under various conditions. It is also unknown what pattern fit indices performance takes under harsh conditions when comparing HLM and CCREM. The major goal of this study is to establish practical guidelines for analyzing cross-classified multilevel data structures by investigating what conditions cause estimation problems from a comprehensive perspective. The specific research questions (RQ) are as follows:

RQ 1. When a crossed factor is ignored, to what degree does the accuracy of the estimates change depending on the simulation conditions?

RQ 2. Are there interaction effects among simulation conditions on estimation bias?

RQ 3. How accurately do relative fit indices detect the correct model between HLM and CCREM?

## Method

Based on the research questions above, a Monte Carlo simulation was conducted. The procedures of the simulation study involved generating population data, analyzing research models under each simulation condition, and evaluating the results based on the evaluation criteria. The datasets were generated by SAS 9.4 (SAS Inc., 2014). To analyze CCREM and HLM, the PROC MIXED procedure in SAS was utilized, while to allow the use of CCREM, a full maximum-likelihood (FML) estimation was employed.

### Data Generation and Analysis

To generate a population model, a two-way CCREM was set with two crossed factors. The two factors were labeled as row and column factors in this study. The population model is shown in Equations 1 and 2:

(1) $$\begin{array}{r} \text{Level }1:Y_{ijk}=\;\pi_{0jk}+\pi_{1jk}X_{ijk}+\;e_{ijk} \end{array}$$

(2) $$\begin{array}{r} \text{Level }2:\pi_{0jk}=\gamma_{000}+\;\gamma_{010}W_{k}+\;\gamma_{020}Z_{j}+\;b_{0j0}+\;c_{00k}\\ \;\pi_{1jk}=\gamma_{100}. \end{array}$$

The subscripts *i*, *j*, and *k* represent the individual, group *j*, and group *k*, respectively. For the sake of convenience, group *j* refers to a specific entity of column factor, group *k* refers to a specific entity of row factor, and *i* represents individuals. There is one outcome variable (*Y*_*ijk*_) affected by each of the three covariates: a predictor for the individual level (*X*_*ijk*_), a predictor for the column factor level (*Z*_*j*_), and a predictor for the row factor level (*W*_*k*_). π_0*jk*_ and π_1*jk*_ in the level 2 model are from the level 1 model; π_0*jk*_ is a random coefficient, and π_1*jk*_ is a fixed coefficient. γ_100_, γ_010_, and γ_020_ are the coefficients of the three predictors. The intercept (γ_000_) was fixed to 100. *b*_0*j*0_ and *c*_00*k*_ were the error term from the two crossed factors. The variance of *e*_*ijk*_ was set at 1.

After the population model was generated, the impact of ignoring one of the crossed factors by comparing an appropriate modeling (CCREM) vs. an inappropriate modeling (HLM) was investigated. The CCREM analysis yielded the outcome variable (*Y*_*ijk*_), one individual level predictor (*X*_*ijk*_), and two cross-classified level predictors (*W*_*k*_ and *Z*_*j*_). This analysis model corresponds to the population model, as shown in Equations 1 and 2. The effect of individual level predictors was set as a fixed effect across the cross-classified factors. Four fixed effects and random effects were estimated in the analysis of CCREM.

For the HLM analysis, one of the cross-classified factors from CCREM was ignored, and only the row factor was considered as a level 2 data structure. The analysis model is shown in Equations 3 and 4:

(3) $$\begin{array}{r} \text{Level }1:Y_{ij}=\;\beta_{0j}+\beta_{1j}X_{ij}+\;\beta_{2j}Z_{ij}+\;r_{ij} \end{array}$$

(4) $$\begin{array}{r} \text{Level }2:\beta_{0j}=\gamma_{00}+\gamma_{01}W_{j}+\;u_{oj}\\ \;\beta_{1j}=\gamma_{10}\\ \;\beta_{2j}=\;\gamma_{20}. \end{array}$$

The subscripts *i* and *j* represent the individual and the cluster. There is one outcome variable (*Y*_*ij*_), one cluster level predictor (*W*_*j*_), and two individual-level predictors (*X*_*ij*_ and *Z*_*ij*_) with normal distribution following a mean of 50 and a standard deviation of 10. Assuming *i* and *j* represent a specific individual and a specific entity of the row factor, *W*_*j*_ would be the remaining predictor of the row factor, which was *W*_*k*_ in the CCREM in Equation 2. In addition, *Z*_*ij*_ is the ignored column factor predictor, which was *Z*_*j*_ in the CCREM in Equation 2. β_0*j*_, β_1*j*_, and β_2*j*_ in the level 2 model are from the level 1 model. β_0*j*_ is a random coefficient, and β_1*j*_ and β_2*j*_ are fixed coefficients. γ_00_ is an intercept, and γ_01_ is a coefficient of a cluster-level predictor, while γ_10_ and γ_20_ are the coefficients of individual-level predictors that have no random parts varying across the level 2 clusters. Finally, *r*_*ij*_ and *u*_*oj*_ are error terms at the individual and cluster levels. Four fixed effects (γ_00_, γ_01_, γ_10_ and γ_20_) and two random effects were estimated.

### Simulation Conditions

As shown in Table 1, six conditions were manipulated: the magnitude of coefficient (0.20, 0.50, and 0.80), the number of feeders (2, 4, and 6), the correlation between the level 2 residuals, which are *b*_0*j*0_ and *c*_00*k*_ in Equation 2 (0 and 0.40), the number of groups of each cross-classified factor (30 and 50), the average number of individuals sampled from the column factor (20 and 40), and the intra-unit correlation coefficient (0.05 and 0.15). A total of 144 (3 × 3 × 2 × 2 × 2 × 2 = 144) conditions were involved in the current study, with 500 replications for each condition. All of the values for each condition were based on previous studies.

**Table 1:** Simulation conditions.

**Conditions**	**Details**
Magnitude of coefficients	0.2 (small), 0.5 (medium), 0.8 (large)
Number of feeders	2, 4, 6
Correlation between the level 2 residuals	0 (no relations), 0.4 (correlated)
Number of groups	30 (small), 50 (large)
Average group size of individuals	20 (small), 40 (large)
Intra-unit class correlation	0.05 (small), 0.15 (moderate)

#### The Magnitude of Coefficient

In the current study, the detailed conditions of the magnitude of coefficient were set as 0.20, 0.50, and 0.80, denoting small, moderate, and large effect sizes, respectively (Cohen, 1988). In previous studies, a moderate effect size was used for all the predictors (Meyers and Beretvas, 2006; Luo and Kwok, 2009; Ye and Daniel, 2017). However, in educational research, a small effect size is common (Spybrook, 2008). To include realistic conditions, small to large effect sizes have been considered in the current study as the magnitude of coefficient.

#### The Number of Feeders

**Figure 1:**
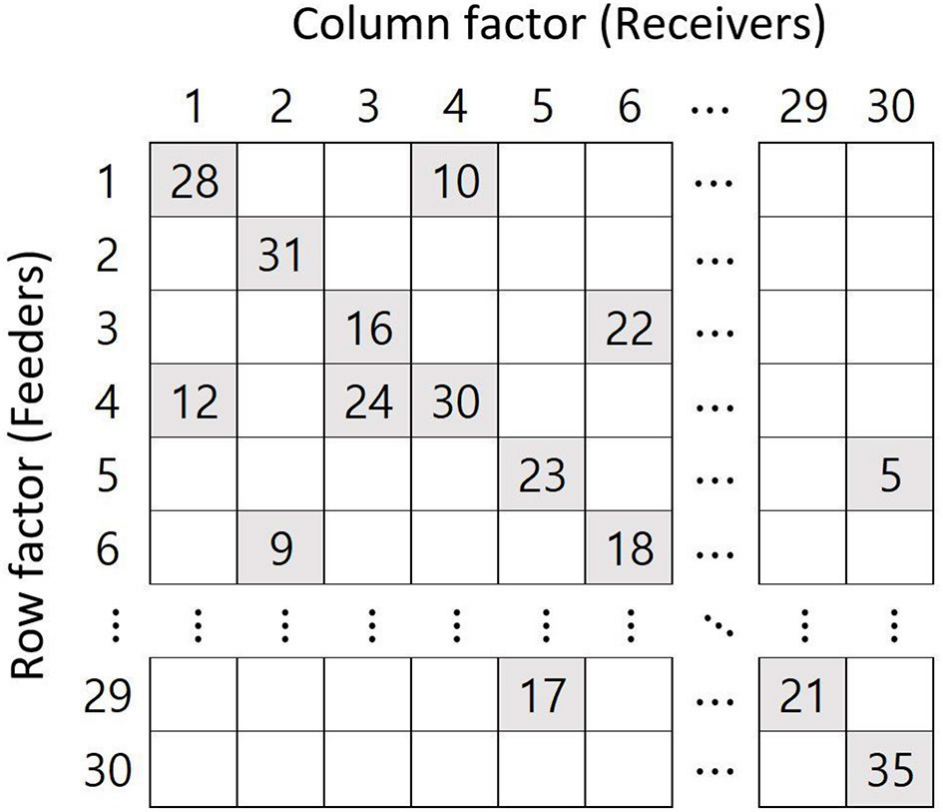
Example of the two-feeder structure when the number of cross-classified factors is 30 and the number of samples per factor is 40.

The structures of cross-classification affect the results (Luo and Kwok, 2009). Generally, a structure of feeders implies a cross-classified data structure. Figure 1 shows a two-feeder structure where the row and column factors indicate feeders and receivers (e.g., neighborhood and school), respectively. Here, each school receives students from two randomly selected neighborhoods, the feeder neighborhoods. For example, for the first column (school 1), there are two feeders, rows 1 and 4 (neighborhoods 1 and 4). The current study sets the number of feeders at 2, 4, and 6, while most previous studies have set the number of feeders as 2 or 3 (Meyers and Beretvas, 2006; Shi et al., 2010; Ye and Daniel, 2017). Wallace (2015) varied the number of feeders to 2 and 4, and Lee and Hong (2019) varied them from 2 to 6. The larger the number of feeders, the more individuals belonging to a *j*th cluster are randomly distributed to several *k*th clusters.

#### The Correlation Between Level 2 Residuals

Generally, there are relationships between the residuals of level 2 crossed factors (Meyers and Beretvas, 2006; Shi et al., 2010), shown in Equation 2 as *b*_0*j*0_ and *c*_00*k*_. For example, students from an elementary school in a high-income area are likely to go to a secondary school in a high-income area, demonstrating that some patterns may be related to a combination of cross-classified factors. For the current study, two conditions of correlation coefficient were used. One is no correlation between the residuals of two types of schools, and the other is a 0.40 correlation between the residuals. The number 0.40 was chosen based on previous research (Meyers and Beretvas, 2006).

#### The Number of Groups

Based on previous studies, the number of groups of each cross-classified factor was set at 30 or 50. Some researchers have used 30 and 50 as the number of groups of cross-classified factors **(**Meyers and Beretvas, 2006; Ye and Daniel, 2017), but in Luo and Kwok (2009), the number of two cross-classified factors was 20 and 50. Although a minimum sample of 50 is recommended for level 2 entities **(**Maas and Hox, 2002**)**, in reality, the number of schools, which is the number of groups in level 2, rarely surpasses 30 **(**Meuleman and Billiet, 2009).

#### The Average Group Size of Individuals

The average number of individuals sampled from the column factor was set at 20 or 40, which means that the group sizes were randomly generated with a mean of 20 or 40. Researchers have insisted that at least 20 **(**Hox, 1998) or 30 individuals **(**Kreft and De Leeuw, 1998) are required for multilevel analysis. Forty was set as the large number of individuals because it is rare for there to be more than 40 students in one class in educational contexts. The number of individuals in each of the groups was randomly generated so that the individuals were drawn from a normal distribution that had a mean of 20 or 40 with a standard deviation of 2. For example, if the number of students in a school was set at 40, the number of students in each school was distributed around 40.

#### The Intra-unit Correlation Coefficient (IUCC)

The IUCC is a ratio of the upper-level variance from the two membership sources out of the total variance when there are two or more membership factors. Meyers and Beretvas (2006) stated that the IUCC values range from 0.009 to 24%, while Wallace (2015**)** set the IUCC conditions at 7 and 13%. In the current study, conditions were set at 5% (small) and 15% (moderate). The IUCC calculation formula is shown in Equation 5:

(5) $$\begin{array}{r} \rho_{jk}=\frac{\tau_{0j0}+\;\tau_{00k}\;}{\tau_{0j0}+\;\tau_{00k}\;+\;\sigma^{2}}. \end{array}$$

The formula ρ_*jk*_ denotes the IUCC when the subscripts *j* and *k* represent two cross-classified factors, while τ_0*j*0_ is the variance of the random effect associated with the factor *j*, and τ_00*k*_ is the variance of the random effect associated with the factor *k*. The variance originating from level 1 individuals (σ^2^) was fixed at 1.0, and the variances of the cross-classified factors (τ_0*j*0_ and τ_00*k*_) were set to be the same value. Referring to Beretvas and Murphy (2013), the variances of the cross-classified factors were set at 0.0556 for the small IUCC and at 0.2143 for the moderate IUCC.

### Evaluation Criteria

There are two types of relative bias to be investigated: parameter estimates bias and SE bias. The parameter bias is calculated as shown in Equation 6, and the SE bias is calculated as shown in Equation 7:

(6) $$\begin{array}{r} B\left(\overset{}{{\bar{\hat{\theta }}}_{r}}\right)=\;\frac{\;\overset{}{{\bar{\hat{\theta }}}_{r}}-\theta \;}{\theta } \end{array}$$

(7) $$\begin{array}{r} B\left({ŝ}_{{\hat{\theta }}_{r}}\right)=\frac{\;{\overset{}{\bar{ŝ}}}_{{\hat{\theta }}_{r}}-\;s_{{\hat{\theta }}_{r}}^{*}\;}{s_{{\hat{\theta }}_{r}}^{*}}. \end{array}$$

θ is the population value set for each condition, ${\hat{\theta }}_{r}$ is the *r*th estimate in the 500 replications, and ${\overset{\bar{}}{\hat{\theta }}}_{r}$ is the mean of the estimates across replications. Where *R* (1, 2, …, *r*) represents the number of replications, the parameter bias is the portion of the difference between the mean of the estimates across replication (${\overset{}{\bar{\hat{\theta }}}}_{r}$) and the population value of estimate (θ) out of the population value of the estimate (θ). Where $s_{{\hat{\theta }}_{r}}^{*}$ is the empirical value standard error that is used as the population value and ${\overset{\bar{}}{ŝ}}_{{\hat{\theta }}_{r}}$ is the mean of the standard error across replications, SE bias is the portion of the difference between the mean of the SE across replication (${\overset{}{\bar{ŝ}}}_{{\hat{\theta }}_{r}}$) and the population value of the SE ($s_{{\hat{\theta }}_{r}}^{*}$) out of the population value of SE ($s_{{\hat{\theta }}_{r}}^{*}$). The acceptable amount of parameter bias is less than 0.05, i.e., $|B\left({\hat{\theta }}_{r}\right)|<$ 0.05, and the acceptable amount of SE bias is less than 0.10, i.e., $|B\left({ŝ}_{{\hat{\theta }}_{r}}\right)|<$ 0.10 (Hoogland and Boomsma, 1998).

An analysis of variance (ANOVA) was conducted focusing on when either the parameter bias or the SE bias failed to meet the evaluation criteria. The bias criteria were used as a dependent variable, and the six conditions were used as factors for ANOVA. For the effect size, partial eta-squared ($\eta_{p}^{2}$) were computed. The guideline to interpret the effect size stipulated the values of 0.010, 0.059, and 0.138, denoting small, medium, and large effect sizes, respectively.

For the information criteria, the Akaike information criterion (AIC) (Akaike, 1973) and the Bayesian information criterion (BIC) (Schwarz, 1978) were used. These criteria compare competing models when the compared models are not nested (Gurka, 2006; Whittaker and Furlow, 2009; Beretvas and Murphy, 2013). The equations for AIC and BIC are in Equations 8 and 9 below:

(8) $$\begin{array}{r} AIC=-2LL+2q. \end{array}$$

(9) $$\begin{array}{r} BIC=-2LL+In\left(N\right)q. \end{array}$$

While −2*LL* (−2 log-likelihood) denotes the deviance statistic, *q* means the number of parameters estimated, and *N* denotes the number of level 1 samples. The smaller values indicate a better fit. For the current study, the rate of detecting the correct model is presented for CCREM with AIC and BIC.

## Results

CCREM and HLM were analyzed for each condition, and the results were represented as follows. First, the biases of the fixed effects were reported. In 144 conditions, the parameter biases and the SE biases of fixed effect estimates of the two models were compared. Second, the biases of the random effects were reported. Similarly, the parameter bias and the SE bias of the two models were compared. For the bias criteria, an ANOVA was conducted to detect significant influences when either the parameter bias or the SE bias was over the acceptable criteria. Last, the percentages of the information criteria for choosing the correct model (CCREM) were reported.

### Parameter Bias and SE Bias for Fixed Effects

All of the parameter biases for the fixed effects results were near zero, which means there was no substantial parameter bias of the estimates in the incorrect model (HLM) when the criterion was less than absolute 0.05. Meanwhile, some of the SE biases for the fixed effects did not meet the acceptable SE bias criterion of less than absolute 0.10 (Hoogland and Boomsma, 1998). Notably, the SE of the intercept and the predictor of both cross-classified factors were underestimated.

#### The Intercept

**Figure 2:**
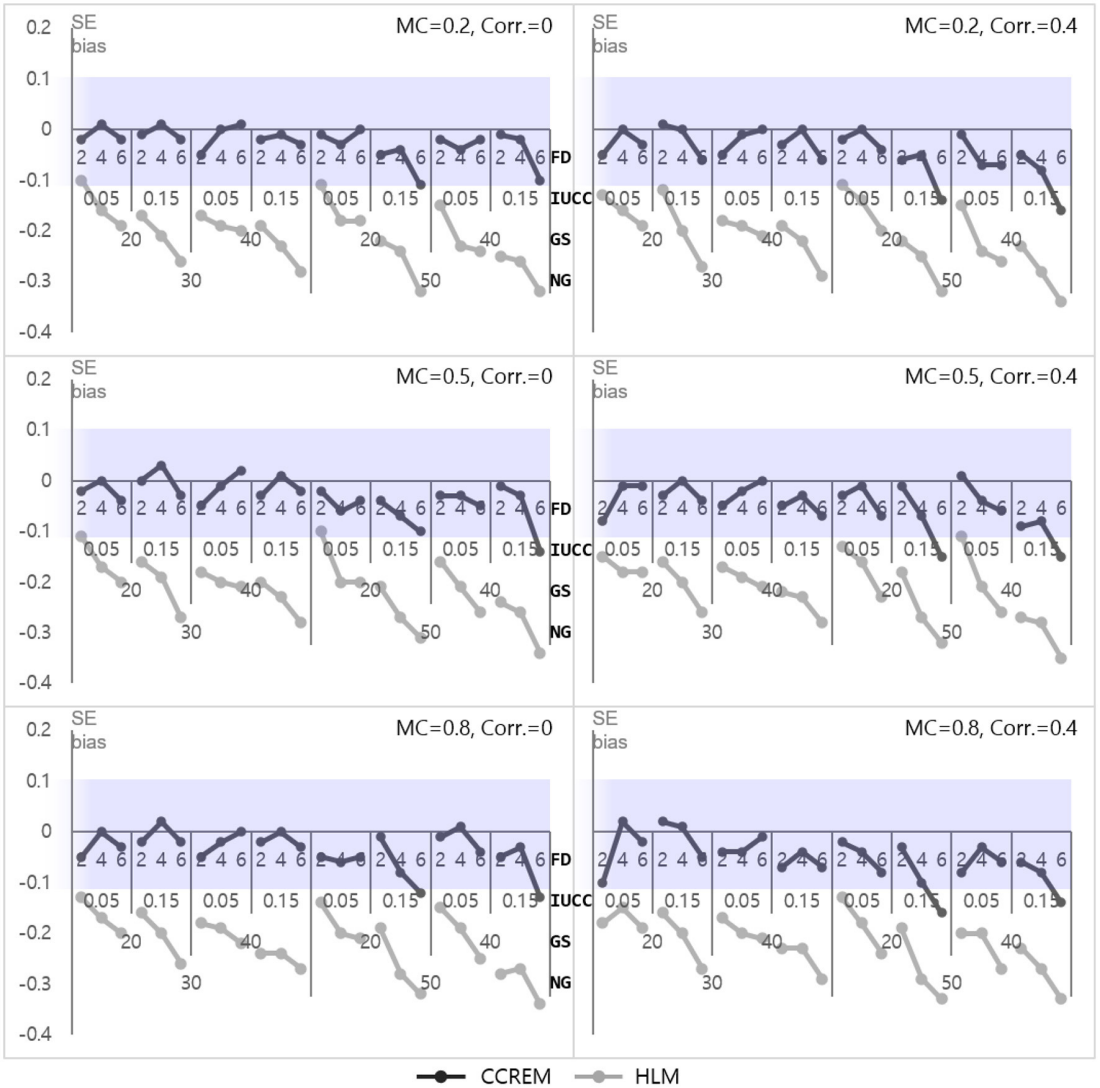
Standard error bias of the intercept. *Notes*. The shaded area indicates the acceptable range. FD, number of feeders; IUCC, intra-unit correlation coefficient; GS, average group size of individuals sampled from the row factor, NG, number of groups of each cross-classified factor; MC, magnitude of coefficients; Corr., correlation between the level 2 residuals.

The parameter biases of the intercepts for both models (γ_000_ for CCREM and γ_00_ for HLM) were below the absolute 0.10, at almost zero. The SE bias for CCREM ranged from −0.160 to 0.030 (M = −0.04, SD = −0.04) and for HLM ranged from −0.35 to −0.10 (M = −0.22, SD = 0.06), reflecting that most of the estimates exceeded the acceptable level, as shown in Figure 2. Since some SE bias values fell outside the acceptable range, an ANOVA was conducted for SE biases to find the source of the differences. For CCREM, the main effect of the number of feeders ($\eta_{p}^{2}\;=$ 0.026), the IUCC ($\eta_{p}^{2}=$ 0.014), and the number of groups ($\eta_{p}^{2}=$ 0.042) showed a small effect size. The interaction effect of the number of groups × the IUCC ($\eta_{p}^{2}=$ 0.017), the number of groups × the number of feeders ($\eta_{p}^{2}=$ 0.026), and the IUCC × the number of feeders ($\eta_{p}^{2}=$ 0.025) showed a medium effect size on the SE bias. Other effect sizes were smaller than 0.005. For HLM, a two-way interaction effect of the number of groups × the IUCC ($\eta_{p}^{2}=$ 0.011), the main effects of the number of groups ($\eta_{p}^{2}=$ 0.026), the average group size ($\eta_{p}^{2}=$ 0.030), the IUCC ($\eta_{p}^{2}=$ 0.114), and the number of feeders ($\eta_{p}^{2}=$ 0.122) were also found to have effects on the SE bias. The main effect of the feeders and IUCC had medium to large effect sizes, and the main effect of the number of groups and average group size had small to medium effect sizes.

#### The Predictor for the Individual Level (X)

The parameter biases of predictors for the individual level (γ_100_ for CCREM and γ_10_ for HLM) were acceptable in all conditions. The SE bias for CCREM ranged from −0.06 to 0.10 (M = 0.001, SD = 0.03) and for HLM ranged from −0.05 to 0.10 (M = 0.001, SD = 0.03). No bias surpassed the criteria for both analysis models.

#### The Predictor for the Row Factor (Z)

**Figure 3:**
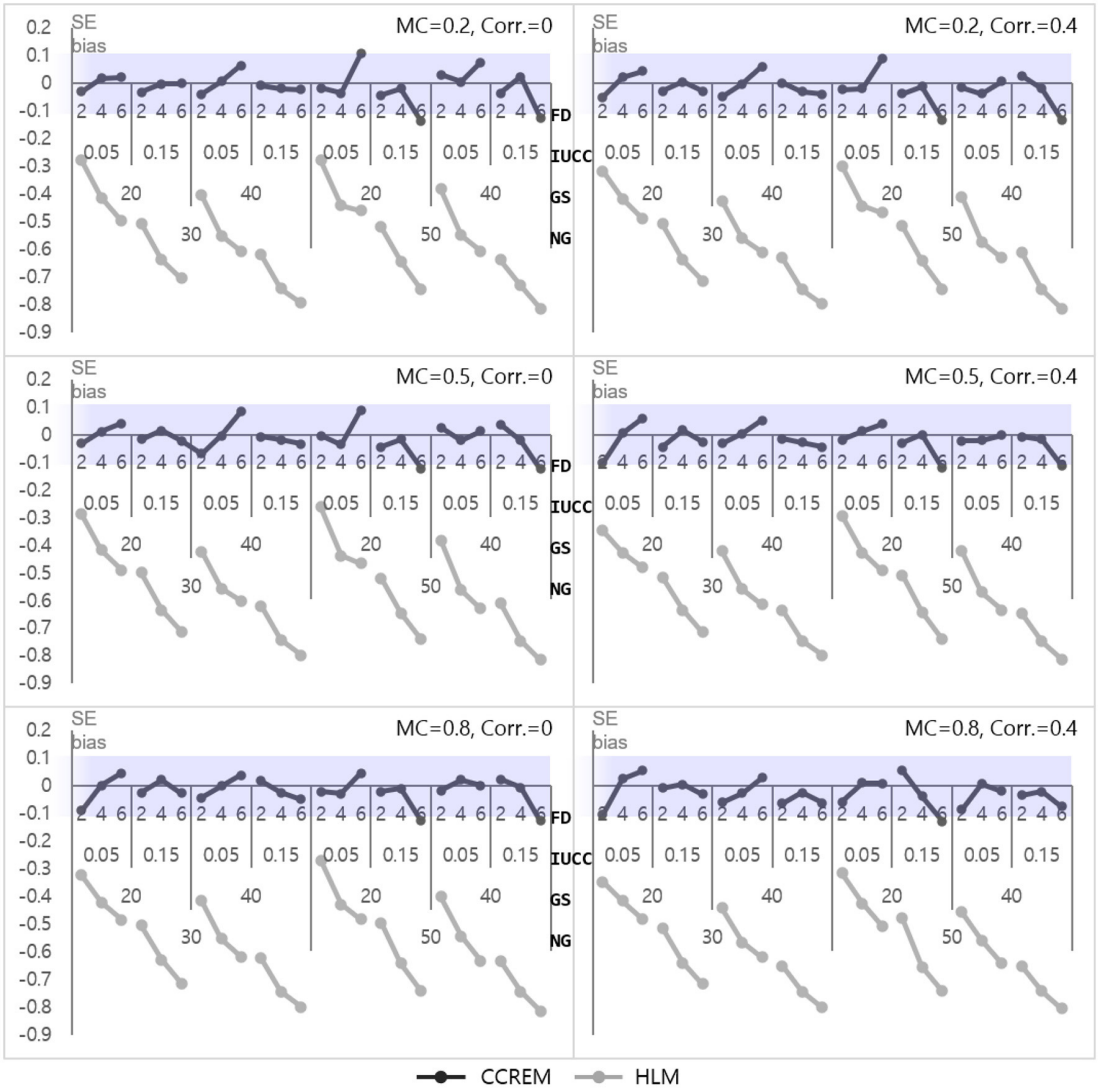
Standard error bias of fixed effect estimates for predictor for the row factor. *Notes*. The shaded area indicates the acceptable range. FD, number of feeders; IUCC, intra-unit correlation coefficient; GS, average group size of individuals sampled from the row factor; NG, number of groups of each cross-classified factor; MC, magnitude of coefficients; Corr., correlation between the level 2 residuals.

The parameter bias of Z, the predictor for the row (γ_010_ for CCREM and γ_20_ for HLM), was near zero, indicating it was acceptable for all conditions. The SE bias for CCREM ranged from −0.14 to 0.11 (M = −0.02, SD = 0.05), and a few values did not meet the criteria. The SE bias for HLM ranged from −0.81 to −0.26 (M = −0.58, SD = 0.15), and most of these values did not meet the criteria of |0.10|, as shown in Figure 3. An ANOVA was conducted for the SE biases of CCREM and HLM. For CCREM, there was a two-way interaction effect for the IUCC × the number of feeders ($\eta_{p}^{2}=$ 0.042) and the number of groups × the number of feeders ($\eta_{p}^{2}=$.011), and the main effect of the IUCC ($\eta_{p}^{2}=$ 0.013), which showed a small to medium effect size. For HLM, a large effect size was found in the main effects of the number of feeders ($\eta_{p}^{2}=$ 0.668), the IUCC ($\eta_{p}^{2}=$ 0.768), and the average group size of individuals sampled from the row factor ($\eta_{p}^{2}=$ 0.501) on the SE bias. There was a two-way interaction effect for the average group size × the IUCC ($\eta_{p}^{2}=$ 0.013) and a three-way interaction effect for the average group size × the IUCC × the number of feeders ($\eta_{p}^{2}=$ 0.015), which showed a small effect size. In HLM, only the cross-classified factor of the rows was modeled for analysis. The cross-classification of the column factor structure was ignored, and a variable of the column factor in CCREM was considered a variable of the individual level in HLM. This misspecification resulted in substantial biases in the SE of the estimation on the coefficient of the predictor for the column factor in HLM.

#### The Predictor for the Second Group Factor (W)

The parameter biases of W, the predictor for The Predictor for the Second Group Factor (W)the second group factor, ranged from −0.15 to 0.16 (M = 0, SD = 0.02) for CCREM and from −0.19 to −0.17 (M = 0, SD = 0.02) for HLM. The SE bias for CCREM ranged from −0.57 to 0.88 (M = −0.004, SD = 0.15) and for HLM ranged from −0.52 to 0.86 (M = −0.004, SD = 0.15). An ANOVA was conducted on the SE bias for both CCREM and HLM. For CCREM, there was a main effect for IUCC ($\eta_{p}^{2}=$ 0.030), with a small to medium effect size. The other effects had an $\eta_{p}^{2}<$ 0.009. For HLM, the main effect for the IUCC ($\eta_{p}^{2}=$ 0.018) had a small effect size. The rest of the values of effect sizes ($\eta_{p}^{2}$) were smaller than .009 in all conditions.

### Parameter Bias and SE Bias for Random Effects When the Correlation Condition Is Zero

For the condition in which the correlation between the level 2 residuals is 0.40, the population value is not known (Meyers and Beretvas, 2006). Thus, the bias results are reported only when the condition of the correlation was zero.

#### Level 1 Model

**Figure 4:**
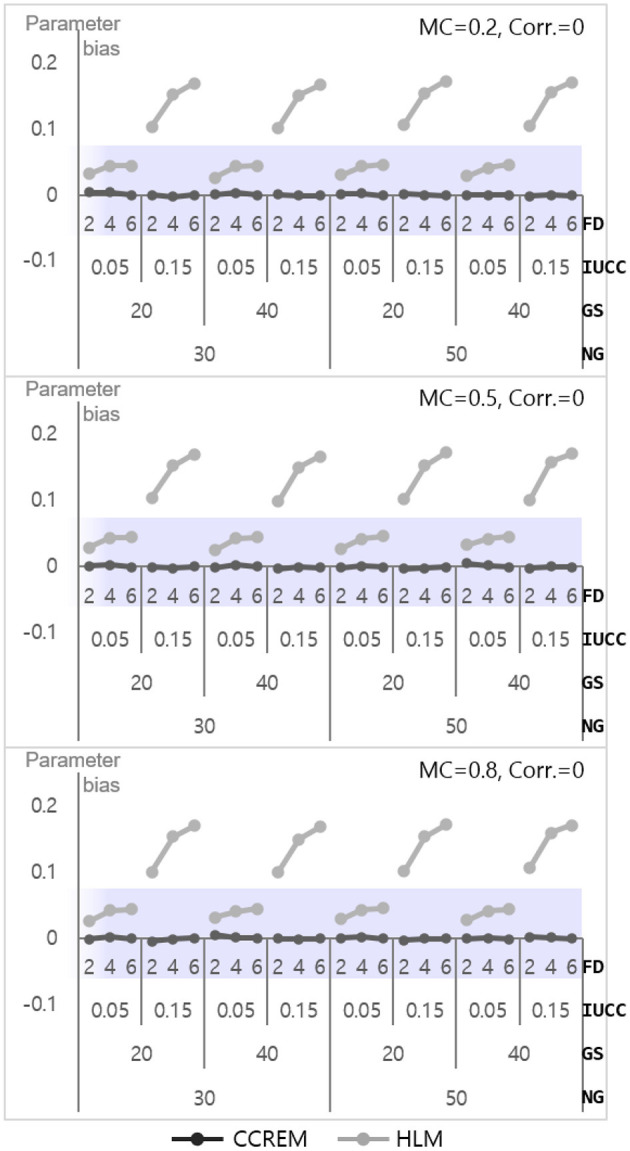
Parameter bias of the level 1 residual with 0 correlation. *Notes*. The shaded area indicates the acceptable range. FD, number of feeders; IUCC, intra-unit correlation coefficient; GS, average group size of individuals sampled from the row factor; NG, number of groups of each cross-classified factor; MC, magnitude of coefficients; Corr., correlation between the level 2 residuals.

**Figure 5:**
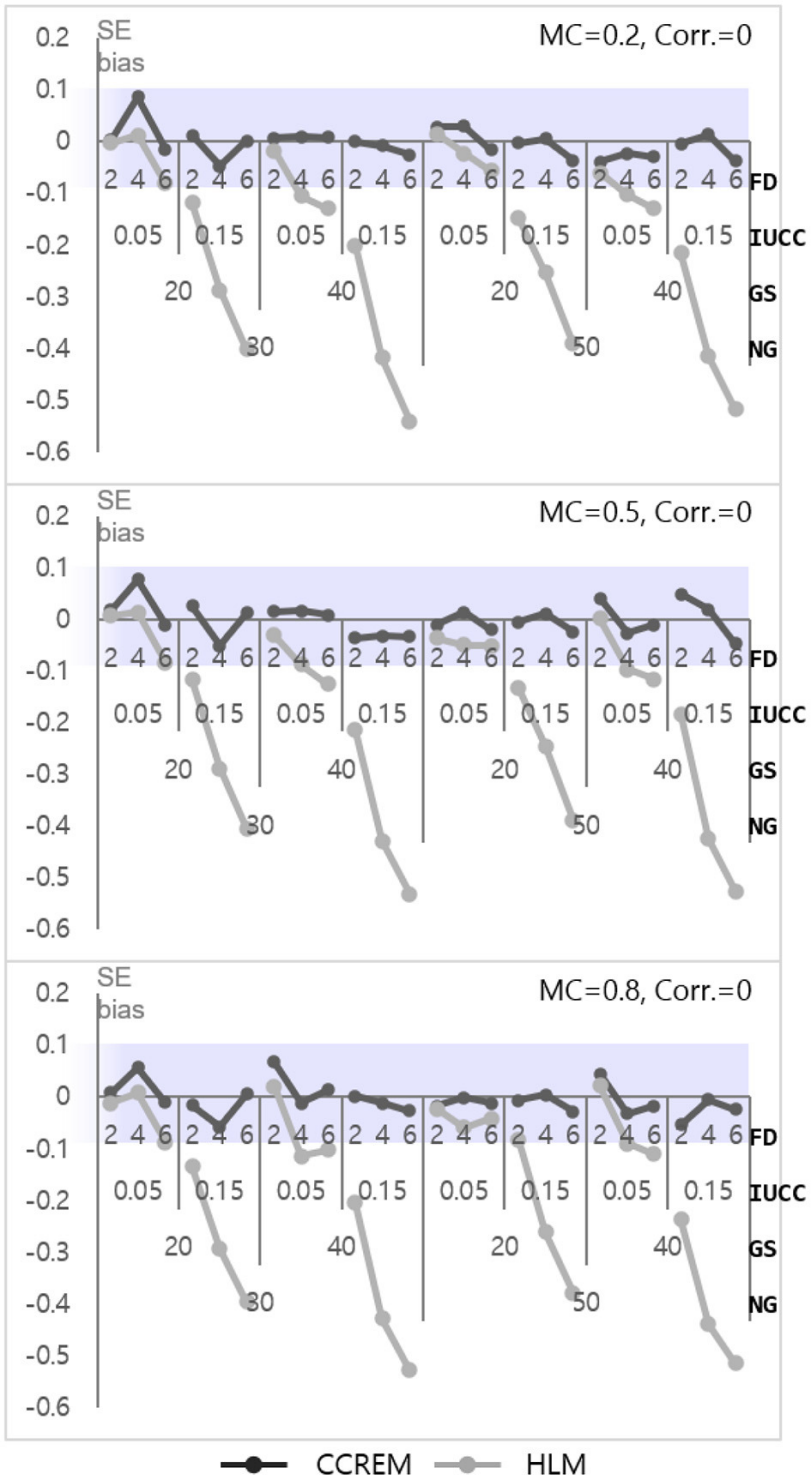
Standard error bias of the level 1 residual with 0 correlation. *Notes*. The shaded area indicates the acceptable range. FD, number of feeders; IUCC, intra-unit correlation coefficient; GS, average group size of individuals sampled from the row factor; NG, number of groups of each cross-classified factor; MC, magnitude of coefficients; Corr., correlation between the level 2 residuals.

As shown in Figure 4, the parameter biases range from −0.01 to 0 (M = 0, SD = 0.001) for CCREM and from 0.02 to 0.17 (M = 0.09, SD = 0.06) for HLM. An ANOVA for parameter bias in HLM was conducted to find the source of the differences. The ANOVA results showed a large effect size for the IUCC ($\eta_{p}^{2}=$ 0.567), a medium effect size for the number of feeders ($\eta_{p}^{2}=$ 0.136), and a small effect size for the two-way interaction of the IUCC × number of feeders ($\eta_{p}^{2}=$ 0.055). However, the effect sizes of the interactions were small in terms of partial eta squared value. The SE biases range from −0.06 to 0.09 (M = −0.003, SD = 0.03) for CCREM and from −0.54 to 0.02 (M = −0.19, SD = 0.17) for HLM. All values of the SE bias were within the acceptable range for CCREM; however, approximately half of the values exceeded the acceptable criterion for HLM, as shown in Figure 5. The results of the ANOVA for the SE bias for HLM showed the main effects, which were the number of feeders ($\eta_{p}^{2}=$ 0.839), the IUCC ($\eta_{p}^{2}=$ 0.941), and the average group size from the row factor ($\eta_{p}^{2}=$ 0.611), all having a large effect size. There were several interaction effects. Focusing on the magnitude of the coefficient, which was one of the main interests of this study, the partial eta squared value of the main effect of the magnitude of the coefficient was below the small effect size. The interaction effects, including the magnitude of coefficient, however, showed small to medium effect sizes. For example, the magnitude of coefficient × the number of feeders ($\eta_{p}^{2}=$ 0.017) showed a two-way interaction effect with small effect size, and the magnitude of coefficient × the average group size × the IUCC ($\eta_{p}^{2}=$ 0.029) showed a three-way interaction effect with small to medium effect sizes.

Only three interaction effects with partial eta squared values were found to be over medium effect size. These were 3 two-way interaction effects: the average group size × the IUCC ($\eta_{p}^{2}=$ 0.254), the average group size × the number of feeders ($\eta_{p}^{2}=$ 0.170), and the IUCC × the number of feeders ($\eta_{p}^{2}=$ 0.622). Considering the average group size × IUCC interaction effect, when the IUCC was small, the SE bias was not problematic, regardless of the average group size, but when the IUCC was moderate, a more severe underestimation happened for the larger average group size. Considering the average group size × the number of feeders interaction effect, when the average group size was small, the SE bias increased consistently, but when the average group size was large, a more severe underestimation happened, especially if the number of feeders was four or more. Finally, considering the IUCC × the number of feeders interaction effect, when the IUCC was small, the SE bias was not problematic, regardless of the number of feeders, but when the IUCC was moderate for a larger number of feeders, the bias became more severe.

#### Level 2 Model

**Figure 6:**
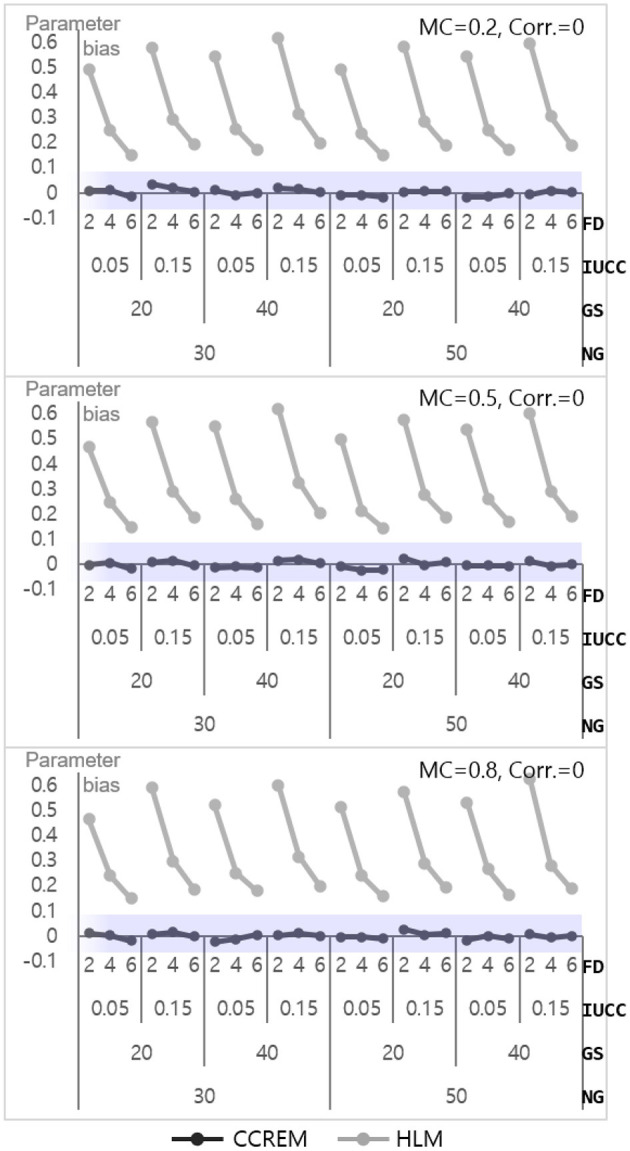
Parameter bias of the level 2 residual with 0 correlation. Notes. The shaded area indicates the acceptable range. FD, number of feeders; IUCC, intra-unit correlation coefficient; GS, average group size of individuals sampled from the row factor; NG, number of groups of each cross-classified factor; MC, magnitude of coefficients; Corr., correlation between the level 2 residuals.

The parameter bias for estimation ranged from – 0.02 to 0.03 (M = 0, SD = 0.01) for CCREM, which means all values met the criterion. However, a serious problem appeared in the parameter bias for HLM, with the values ranging from 14 to 62 (M = 0.34, SD = 0.16). As shown in Figure 6, the biases decreased as the number of feeders increased in HLM. The ANOVA results showed that the main effect of the number of feeders on the accuracy parameter estimates had a large effect size ($\eta_{p}^{2}=$ 0.139). Other effects' $\eta_{p}^{2}$ were less than 0.005. The SE bias for CCREM appeared to range from −0.09 to 0.10 (M = 0.00, SD = 0.04), implying that a minority of the estimates exceeded the acceptable range. For HLM, the values ranged from −0.08 to 0.07 (M = −0.01, SD = 0.03), indicating there was no problem in the SE bias in the level 2 model.

### Parameter Bias and SE Bias for Random Effects When the Correlation Condition Is 0.40

The analysis revealed estimation results only and gave no results about bias when the correlation condition was 0.40 because the population values were unknown when the correlation between the level 2 residuals was 0.40.

#### Level 1 Model

The estimates of CCREM ranged from 0.99 to 1.00 (M = 1.00, SD = 0.002). However, the estimates in HLM were frequently overestimated, and the parameter estimates ranged from 1.02 to 1.17 (M = 1.09, SD = 0.06), in which larger values appeared in the IUCC = 0.15 condition. The standard deviation of CCREM ranged from 0.02 to 0.06 (M = 0.03, SD = 0.01) and that of HLM showed almost the same range and descriptive statistics. The standard deviation decreased as the number of feeders increased.

#### Level 2 Model

Table 2 shows the results of the estimates when the correlation was 0.40. The estimates in CCREM ranged from 0.06 to 0.25 (M = 0.15, SD = 0.09), and those in HLM ranged from 0.07 to 0.46 (M = 0.22, SD = 0.14). The standard deviations in CCREM ranged from 0.01 to 0.10 (M = 0.04, SD = 0.02) and in HLM from 0.02 to 0.14 (M = 0.06, SD = 0.04). The difference in the estimates between CCREM and HLM resulted from ignoring a cross-classified factor; HLM ignored the row factor and only modeled the second group factor. This inappropriate modeling resulted in overestimation in HLM.

**Table 2:** Means and standard deviations of Level 2 residuals when the correlation condition equals 0.40.

				**CCREM**			**HLM**		
				**Feeder**			**Feeder**		
				**2**	**4**	**6**	**2**	**4**	**6**
**MC**	**NG**	**GS**	**IUCC**	***M(SD)***	***M(SD)***	***M(SD)***	***M(SD)***	***M(SD)***	***M(SD)***
0.2	30	20	0.05	0.07 (0.04)	0.06 (0.02)	0.06 (0.02)	0.11 (0.04)	0.08 (0.03)	0.07 (0.02)
			0.15	0.25 (0.10)	0.23 (0.07)	0.22 (0.06)	0.45 (0.14)	0.33 (0.10)	0.29 (0.08)
		40	0.05	0.06 (0.03)	0.06 (0.02)	0.06 (0.02)	0.11 (0.04)	0.08 (0.03)	0.07 (0.02)
			0.15	0.24 (0.08)	0.23 (0.07)	0.22 (0.06)	0.45 (0.13)	0.34 (0.09)	0.30 (0.08)
	50	20	0.05	0.06 (0.03)	0.06 (0.02)	0.06 (0.02)	0.11 (0.03)	0.08 (0.02)	0.07 (0.02)
			0.15	0.25 (0.08)	0.22 (0.05)	0.22 (0.05)	0.44 (0.10)	0.33(0.07)	0.29 (0.06)
		40	0.05	0.06 (0.02)	0.06 (0.02)	0.06 (0.01)	0.11 (0.03)	0.08 (0.02)	0.07 (0.02)
			0.15	0.24 (0.06)	0.22 (0.05)	0.22 (0.05)	0.45 (0.10)	0.33 (0.07)	0.29 (0.06)
0.5	30	20	0.05	0.07 (0.04)	0.06 (0.02)	0.06 (0.02)	0.11 (0.04)	0.08 (0.03)	0.07 (0.02)
			0.15	0.25 (0.10)	0.23 (0.07)	0.22 (0.06)	0.45 (0.14)	0.33(0.10)	0.29 (0.08)
		40	0.05	0.06 (0.03)	0.06 (0.02)	0.06 (0.02)	0.11 (0.04)	0.08 (0.03)	0.07 (0.02)
			0.15	0.25 (0.09)	0.22 (0.07)	0.22 (0.06)	0.46 (0.13)	0.34 (0.09)	0.29 (0.08)
	50	20	0.05	0.07 (0.03)	0.06 (0.02)	0.06 (0.02)	0.11 (0.03)	0.08 (0.02)	0.07 (0.02)
			0.15	0.25 (0.08)	0.22 (0.05)	0.22 (0.05)	0.44 (0.10)	0.33 (0.07)	0.29 (0.06)
		40	0.05	0.06 (0.02)	0.06 (0.02)	0.06(0.01)	0.11 (0.03)	0.08 (0.02)	0.07(0.02)
			0.15	0.24 (0.06)	0.22 (0.05)	0.22 (0.05)	0.46 (0.10)	0.33 (0.07)	0.29 (0.06)
0.8	30	20	0.05	0.07 (0.04)	0.06 (0.02)	0.06 (0.02)	0.11 (0.04)	0.08 (0.03)	0.07(0.02)
			0.15	0.25 (0.10)	0.23 (0.07)	0.22 (0.06)	0.44 (0.14)	0.33 (0.10)	0.29 (0.08)
		40	0.05	0.06(0.03)	0.06 (0.02)	0.06 (0.02)	0.11 (0.04)	0.08 (0.03)	0.07 (0.02)
			0.15	0.24 (0.09)	0.22 (0.06)	0.22 (0.06)	0.46 (0.13)	0.33 (0.09)	0.29 (0.08)
	50	20	0.05	0.06 (0.03)	0.06(0.02)	0.06 (0.02)	0.10 (0.03)	0.08 (0.02)	0.07 (0.02)
			0.15	0.25 (0.07)	0.22 (0.05)	0.22 (0.05)	0.44 (0.10)	0.33(0.07)	0.29 (0.06)
		40	0.05	0.07 (0.02)	0.06 (0.02)	0.06 (0.01)	0.12 (0.03)	0.09 (0.02)	0.07 (0.02)
			0.15	0.24 (0.06)	0.22 (0.05)	0.22(0.05)	0.45 (0.10)	0.33 (0.07)	0.29 (0.06)

*Notes. Numbers in parentheses are standard deviations. M, means; SD, standard deviations; MC, magnitude of coefficients; COR, correlation between the level 2 residuals; NG, number of groups of each cross-classified factor; GS, average group size of individuals sampled from the first group factor; IUCC, intra-unit correlation coefficient, Feeder: number of feeders*.

Numbers in parentheses are standard deviations.

### Information Criteria

AIC and BIC were used to investigate whether these information criteria indicate that the CCREM is the correct model. The proportions of the 500 replications for 144 conditions were summarized. The proportions of identification of the correct model were examined.

For all conditions, BIC perfectly identified CCREM as the better model. This result is consistent with previous studies which showed that BIC outperformed AIC in model selection **(**Bickel et al., 1992; Zhang, 1993; Raftery and Zheng, 2003; Acquah, 2010) In the case of AIC, the accuracy of identification differed according to the condition. The percentage of correct identification for six feeders was 100%, while the average of the percentage decreased as the number of feeders decreased until the percentage for two feeders fell to 97%. The average percentage for correct identification was 98.51% when the IUCC was 0.05 and 99.98% when the IUCC was 0.15.

The ANOVA results revealed distinct effects on AIC performance. The main effect of the number of feeders ($\eta_{p}^{2}=$ 0.022) had a small to medium effect size, while the interaction effect of the average group size × the number of feeders ($\eta_{p}^{2}=$ 0.014) and that of the average group size× the IUCC× the number of feeders ($\eta_{p}^{2}=$ 0.013) showed a small effect size.

## Discussion

The present simulation study compared the performance of estimates between CCREM and HLM. A Monte Carlo simulation study was conducted in which the data were generated with two cross-classified factors. Six conditions were manipulated: the magnitude of coefficients, the number of feeders, the correlation between the level 2 residuals, the number of groups of each cross-classified factor, the average group size of the individuals sampled from the row factor, and the IUCC. Overall, four fixed effects and two random effects of both analysis models were summarized for each condition and compared with the counterpart model's estimates. An ANOVA was conducted when either the parameter bias or the SE bias fell outside the criteria. In addition, the proportion of identification of the correct model, AIC and BIC, were presented. The results of this study are summarized below and their implications are discussed.

First, for the fixed effects, there were no problems in the parameter or SE biases in CCREM. In HLM, however, the SE bias exceeded the acceptable range in the case of the intercept and the predictor of the first cross-classified factor. As in Meyers and Beretvas (2006), the results showed that the SE was underestimated when one of the crossed factors was ignored in an inappropriate model. According to Raudenbush and Bryk (2002), the misestimated SE can occur when a researcher fails to consider the homogeneity among individuals in multilevel data, and the underestimation of the SE can cause the inflation of a Type 1 error when the cross-classified data structure is not considered. Generally, the SE of fixed effects estimates at the upper level is underestimated. Consistent with Lai (2019), Luo and Kwok (2009, 2012), and Meyers and Beretvas (2006), the SE bias increased with an increased number of feeders or increased IUCC.

The average group size of individuals sampled from the first cross-classified factor was also found to have an effect on the SE of the predictor of the first cross-classified factor. The larger the group size became, the more severe the SE biases became. In terms of the intercept and estimates of the predictor of the first cross-classified factor, the SE biases increased as the group size became larger because the within-group homogeneity is ignored due to misspecification (Meyers and Beretvas, 2006). Known as the design effect, this outcome means that the estimation bias increases as the group size increases (Kalton, 2020). The IUCC and the number of feeders were found to have effects on the SE bias. The degree of underestimation of the SE became larger when the IUCC and the number of feeders increased. In particular, the number of feeders was found to have an effect on the SE bias. The magnitude of coefficients, however, had no meaningful effect on the SE biases for fixed effects.

Second, for the random effects, the estimates of the level 1 and level 2 models were affected by the model misspecification. The parameter bias and the SE bias from CCREM appeared to be in the acceptable range for both the lower and upper levels. On the other hand, in HLM, the parameter estimates of the level 1 and level 2 models were overestimated, and the standard errors of the level 1 model were underestimated. SE biases corresponding to the level 1 residual model were underestimated when the correlation between the level 2 residuals was zero, as in Luo and Kwok (2012). For the estimation of the level 1 component, the results show that when a crossed factor is ignored, the estimation of the lower level is greatly affected. The severity mainly depends on the data structure, i.e., the IUCC and the number of feeders. In the level 1 model, there were medium-sized interaction effects only on SE bias when ignoring cross-classified structures. By considering various simulation factors simultaneously that previous studies evaluated separately, this study investigated the interaction effects as well as the main effects of the factors. If each factor is tested separately, simulation results based solely on main effects can be misleading. In the level 2 model of HLM, the number of feeders had a large effect on the parameter bias, and the biases became larger as the number of feeders decreased.

Last, for the comparison of the performance of fit indices, BIC outperformed AIC. While BIC identified the correct model in all simulation conditions, the percentage of correct identification differed by condition in AIC, where more feeders correlated positively with better accuracy. This result was consistent with previous studies which found that BIC outperforms AIC (Bickel et al., 1992; Zhang, 1993; Raftery and Zheng, 2003; Acquah, 2010).

This study offers the following implications. First, it is noticeable that the impact of misspecification was examined by focusing on the magnitude of coefficients and the number of feeders. Only some interaction effects, including the magnitude of coefficients, showed a small effect size concerning the SE bias of the level 1 model. The number of feeders, however, had a substantial effect on all of the parameter and SE biases. This result supports findings from previous research that the number of feeders could be the cause of estimation bias. Ignoring a crossed factor in cross-classified data structure resulted in problems for estimation, and it is recommended that researchers consider the feeder structure when the data have a cross-classified data structure, especially in cases where the research interest is focused on level 2 estimates. Luo and Kwok's (2009) research model design focused on the CCREM structure that occurred at the top or intermediate levels in the case of three-level data. In the current study, the SE bias of fixed effects did not depend on the magnitude of coefficients. However, the SE bias of level 1 random effects depended on some interaction effects, for example, the magnitude of coefficient × the number of feeders. A researcher designing a study to obtain an appropriate sample size for the use of CCREM may wonder if the effect size does not affect the bias, for example, whether the magnitude of coefficients affects the estimation or not. In addition, the number of feeders, considered one of the conditions in Luo and Kwok (2009), was 5, 25, and 45; in such a large variation in conditions, the effect of the condition easily appears. In this study, the range of the feeder condition was smaller, yet were appropriate for investigating how much of the feeder condition affects the estimation bias. This finding can offer useful information for applied researchers.

Second, the results of this study are likely to help in choosing an analysis model. In terms of the fit indices, the hit rate of BIC was generally better than that of AIC, and there was a tendency toward higher discrepancies in the hit rate as the number of feeders decreased. These findings are significant for researchers selecting between HLM and CCREM for an analysis model. For instance, if the dependent variable were the academic achievement of third-year high school students, high school membership may influence the dependent variable much more than middle school membership. In this case, there are the more important cross-classified factor and the less important cross-classified factor. CCREM is not always an available option, and it is not always possible to obtain preplanned cross-classified data with rich conditions. In such cases, there may be situations in which a researcher should choose whether to consider the less important cross-classified factor.

## Data Availability Statement

The raw data supporting the conclusions of this article will be made available by the authors, without undue reservation.

## Author Contributions

SK planned and carried out the simulations, contributed to the interpretation of the results, and took the lead in writing the manuscript. YJ designed the study, developed the theoretical formalism, and drafted the manuscript. SH supervised the directions of this research and contributed to the interpretation of the results. All authors discussed the results and commented on the manuscript.

## Conflict of Interest

YJ was employed by company Samsung Electronics Leadership Center. The remaining authors declare that the research was conducted in the absence of any commercial or financial relationships that could be construed as a potential conflict of interest.
